# Monthly Summary

**Published:** 1892-04

**Authors:** 


### Monthly Summary.

TO STUDENTS.—You are entering a profession which demands of you not only the actions but the instincts of a gentleman. If you are not possessed of these, it is your duty to acquire them. If your future patrons are to be what any dentist with a spark of ambition desires, then they will be the most refined in the community where he casts his lot. You cannot secure these by carrying into practice the customs that too often prevail in the class-room of a dental college. Some of you are addicted to practices and manners that are far from pleasing. The ethics of gentlemanly conduct seem to be unknown. You go unkempt in the class-room or on the street. You chew tobacco and care little where the spittle is deposited. You do not respect and regard the feelings and comfort of others. You pass your professors with scarcely a nod in recognition of all they do for you at a sacrifice of pleasure and money. Your inattention drives your teachers to think that you are content to go through college, just escape being plucked, and then engage in an advertising business which will forever keep you on a low plane, disgrace your college, and lower your profession. If any of these criticisms apply to you, then set about their correction. The college life of the student will be a pretty sure indication of what his life will be when he enters active practice, therefore if you value your future you will be thankful for these suggestions and take them in as kindly a spirit as they are given.— *Western Dental-Journal.*

---

ACIDITY OF THE STOMACH.—This condition is due to germs, and the cure lies in getting rid of the germs. Germs of fermentation in the stomach, produce first alcohol, then caroblic acid, and then acetic acid. A person troubled with this form of dyspepsia should be careful to take only such articles of food as do not favor the development of germs, and thus starve them out. Another thing to do is to wash the germs out of the stomach while drinking freely of hot water before meals. If food is put into a stomach already sour, of course fermentation will be set up immediately. Some per sons notice that as soon as they eat, their stomachs become sour. The third important thing to do, is to stimulate the stomach to make more gastric juice, which is a natural antiseptic, and prevents fermentation and, also, hastens absorption. The glands may be stimulated by applying hot fomentations to the stomach for half an hour immediately after the close of a meal, or, easier still, by wearing a rubber bag filled with hot water directly over the stomach for half an hour or an hour. Heat is a natural stimulant, and there are no possible ill-effects from its use in this way.—*Good Health.*

